# Editorial: Extracellular vesicles and cell-cell communication in normal cellular processes and cancer

**DOI:** 10.3389/fmolb.2023.1172797

**Published:** 2023-03-14

**Authors:** Abhimanyu Thakur, Zhubo Wei, Huanhuan Joyce Chen

**Affiliations:** ^1^ Pritzker School of Molecular Engineering, University of Chicago, Chicago, IL, United States; ^2^ Ben May Department for Cancer Research, University of Chicago, Chicago, IL, United States; ^3^ Institute of Biosciences and Technology, Texas A&M University, Houston, TX, United States

**Keywords:** cancer, exosome, intracellular communication, stem cell, extracellular vescicles

Cell-cell communication is critical for almost every aspect of cell function. This communication can be facilitated by extracellular vesicles (EVs), which are a group of diverse membrane-enclosed structures created and released by cells and include two main types, exosomes and microvesicles (MVs). Exosomes are approximately 30–100 nm in diameter and are released from cells through endosomal system. MVs are approximately up to 1,000 nm and released from cells *via* budding off from the plasma membrane (Thakur et al., 2017a; van Niel et al., 2018; Thakur et al., 2020a; Thakur et al., 2022a). These EVs are produced by a variety of cell types and contain various cargoes of proteins, lipids, and nucleic acids. EVs may play a crucial role in cell-cell communication because they can act as mediators between cells, allowing them to exchange both materials and information, and cooperate with each other (Thakur et al., 2021).

EVs have been reported to mediate many normal cellular processes such as cell proliferation, differentiation, and migration (Thakur et al., 2017b; Liu et al., 2022). They can also promote the transfer of genetic information and the delivery of therapeutic molecules. In addition, EVs can act as signal mediators that regulate gene expression, angiogenesis, immune responses, and cell metabolism (Thakur et al., 2022b). EV-mediated cell-cell communication is also important for many pathophysiological processes, such as the development of cancer. Cancer cells are known to produce and release EVs that may contribute to each step of cancer development, from initiation, progression, metastasis, to disease relapse and therapy resistance. (Xu et al., 2021). These EVs contain cancer-promoting molecules that can alter the behavior of surrounding cells and microenvironment (Thakur et al., 2022c). For example, cancer cell derived EVs contain oncogenic growth factors, proteins or RNAs which can stimulate the proliferation of neighboring cancer cells. In addition, EVs released by cancer cells contain molecules that can suppress the immune response and protect cancer cells from being eliminated or death (Thakur et al., 2022a; Thakur et al., 2022b).

The ability of EVs to mediate cell-cell communication has also been utilized for cancer therapy, as EVs can be used to deliver therapeutic molecules (Gaurav et al., 2021). For example, EVs from different cell sources have been used for targeted delivery. EVs have been shown to have various anti-tumor effects, such as inducing cell death, promoting tumor regression, and inhibiting tumor growth and metastasis. Additionally, EVs can be used to deliver therapeutic agents such as small interfering RNAs (siRNAs) and microRNAs (miRNAs) to target cells, allowing for the regulation of gene expression and providing a more precise and targeted approach to cancer therapy (Thakur et al., 2022a; Thakur et al., 2022b). Moreover, cancer cell-derived EVs can be used to deliver small interfering RNAs (siRNA) to neighboring cancer cells, which inhibits the expression of cancer-promoting genes. Similarly, EVs can be engineered to deliver drugs to target cancer cells or surrounding non-cancer cells that support the tumor niche (Thakur et al., 2018; Thakur et al., 2020b). These strategies aim to reduce the spread of cancer and to make cancer cells more sensitive to chemotherapy or radiation.

In our present Research Topic of research, different aspects of exosome-based investigation have been included (Jin et. al.; Qiu et. al.; Hayasaka et. al.). Jin et. al. showed the association of lymphoid-restricted membrane protein (LRMP) with immune inflation, which acts as a prognostic biomarker in lung adenocarcinoma (LUAD). LRMP is a protein found in B and T cells, but its role in LUAD is unknown. It is expressed in a regulated manner in these cells, and may influence tumor growth, prognosis, and immune infiltration in LUAD. Further research was needed to understand its role. The authors found that LRMP expression is significantly lower in LUAD tissues and cell lines and is associated with a better prognosis in patients. Overexpression of LRMP decreased proliferation, migration, and invasion in A549 cells, and downregulated oncogenic signaling pathways. Gene set enrichment analysis (GSEA) results showed that immuno-related pathways were enriched in samples with high LRMP. LRMP is also positively correlated with tumor-infiltrating immune cells and their markers, as well as with immune checkpoints. These findings suggest that LRMP may be a tumor suppressor gene and involved in immunotherapy response for LUAD. Further research is needed to validate these findings (Jin et. al.).

In another study, Qiu et. al. demonstrated the diagnostic potential of plasma EVs miR-483-3p and Let-7d-3p for Sepsis. In this study, eleven miRNAs were identified in a discovery cohort of 3 sepsis patients and three healthy controls, with six of these being further validated in a validation cohort of 37 sepsis patients and 25 healthy controls using qRT-PCR. It was found that miR-483-3p and let-7d-3p were elevated in the sepsis group, and their expression correlated with the severity of the disease. Furthermore, a combination of miR-483-3p and let-7d-3p had diagnostic value for sepsis. Bioinformatic analysis and experimental validation also showed that these miRNAs target pathways involved in immune response and endothelial function. This study highlights the potential of EV-miRNAs as biomarkers for sepsis diagnosis, (Qiu et. al.). The advantages of using EVs for disease diagnosis are numerous. First, EVs are relatively easy to obtain and can be collected from a variety of bodily fluids. Second, EVs have a relatively long shelf-life and can be stored for long periods of time. Third, EVs can carry a variety of disease-specific molecules, including proteins, lipids, and nucleic acids, which can be used for diagnosis. Fourth, EV analysis is non-invasive and does not require invasive procedures. Apparently, EV analysis is a relatively conveient and reliable method for disease diagnosis (Thakur et al., 2022a; Thakur et al., 2022b).

Hayasaka et. al. studied the metabolomics of EVs derived from isocitrate dehydrogenase 1 (IDH1)-mutant human colon (HCT116) cells collected *via* semi-automated size exclusion chromatography (SEC). The authors emphasized the importance of EV-isolation and developed several protocols for it. Here, a semi-automated system that combines SEC and high-performance liquid chromatography was developed and tested. It was found to recover EVs more efficiently and non-destructively than ultracentrifugation, making it an ideal method for metabolic analysis. The system was then used to analyze IDH1-mutated HCT116 cells and their EVs. It was found that the IDH mutant cells released significantly more EVs and had a distinct metabolomic profile. Notably, large amounts of 2-hydroxyglutaric acid (2-HG) were detected in both cells and EVs, suggesting that the developed system has potential applications in EV research (Hayasaka et. al.).

In addition to research articles, this Research Topic also includes a few review articles, which comprehensively addressed various crucial aspects of EVs. Szeliski et. al. elaborated the role of EVs as a multicomponent biomarker platform in urinary tract carcinomas (Szeliski et. al.). Chhetri et al. elaborated and talked about the pleiotropic effects of DCLK1 in cancer and cancer stem cells (Chhetri et al.). Zhi et. al. discussed about the research advances and challenges in tissue derived EVs (Zhi et. al.).

In summary, this Research Topic of research articles as well as review articles will be an important source of knowledge for future studies pertaining to EVs as a crucial mediator of cell-cell communication in normal cellular processes and cancer. With clear understanding of their roles in cell-cell communication, new therapeutic strategies can be developed to improve health and to treat diseases including cancer.
